# An Assessment of Perspectives and Concerns Among Research Participants of Childbearing Age Regarding the Health-Relatedness of Data, Online Data Privacy, and Donating Data to Researchers: Survey Study

**DOI:** 10.2196/41937

**Published:** 2023-03-10

**Authors:** Rachele Hendricks-Sturrup, Christine Y Lu

**Affiliations:** 1 Duke-Margolis Center for Health Policy Washington, DC United States; 2 Department of Population Medicine Harvard Pilgrim Health Care Institute and Harvard Medical School Boston, MA United States; 3 Department of Interdisciplinary Health Studies Ohio University Athens, GA United States

**Keywords:** privacy, digital data, research, abortion, women's health, reproductive health, reproductive informatics, digital footprint, family planning

## Abstract

**Background:**

The June 2022 US Supreme Court decision to ban abortion care in *Dobbs v Jackson Women’s Health Organization* sparked ominous debate about the privacy and safety of women and families of childbearing age with digital footprints who actively engage in family planning, including abortion and miscarriage care.

**Objective:**

To assess the perspectives of a subpopulation of research participants of childbearing age regarding the health-relatedness of their digital data, their concerns about the use and sharing of personal data online, and their concerns about donating data from various sources to researchers today or in the future.

**Methods:**

An 18-item electronic survey was developed using Qualtrics and administered to adults (aged ≥18 years) registered in the ResearchMatch database in April 2021. Individuals were invited to participate in the survey regardless of health status, race, gender, or any other mutable or immutable characteristics. Descriptive statistical analyses were conducted using Microsoft Excel and manual queries (single layer, bottom-up topic modeling) and used to categorize illuminating quotes from free-text survey responses.

**Results:**

A total of 470 participants initiated the survey and 402 completed and submitted the survey (for an 86% completion rate). Nearly half the participants (189/402, 47%) self-reported to be persons of childbearing age (18 to 50 years). Most participants of childbearing age agreed or strongly agreed that social media data, email data, text message data, Google search history data, online purchase history data, electronic medical record data, fitness tracker and wearable data, credit card statement data, and genetic data are health-related. Most participants disagreed or strongly disagreed that music streaming data, Yelp review and rating data, ride-sharing history data, tax records and other income history data, voting history data, and geolocation data are health-related. Most (164/189, 87%) participants were concerned about fraud or abuse based on their personal information, online companies and websites sharing information with other parties without consent, and online companies and websites using information for purposes that are not explicitly stated in their privacy policies. Free-text survey responses showed that participants were concerned about data use beyond scope of consent; exclusion from health care and insurance; government and corporate mistrust; and data confidentiality, security, and discretion.

**Conclusions:**

Our findings in light of *Dobbs* and other related events indicate there are opportunities to educate research participants about the health-relatedness of their digital data. Developing strategies and best privacy practices for discretion regarding digital-footprint data related to family planning should be a priority for companies, researchers, families, and other stakeholders.

## Introduction

The June 2022 Supreme Court of the United States ruling on *Dobbs v Jackson Women’s Health Organization* has been an alarming development for many, especially women and families who may experience miscarriage or seek abortion regardless of medical necessity. Digital data today come from a variety of sources, including but not limited to period-tracking apps, electronic health records, and fitness-tracking apps and wearables, that are loosely regulated with respect to personal data privacy and discretion. It is therefore concerning that powerful entities like state law enforcement and large companies may have potentially unfiltered access to sensitive data that are suggestive of female fertility or family planning decisions. Several sources have amplified these concerns, and the future is unknown regarding the personal safety and privacy of women and families of childbearing age with digital footprints who actively engage in family planning, including abortion and miscarriage care [1-6].

The *Dobbs v Jackson* US Supreme Court ruling effectively reversed the 1973 US Supreme Court ruling in *Roe v Wade*, 410 US 113, that conferred on women the right to decisional privacy around abortion and miscarriage care. Prior to and at the time of *Roe v Wade*, the proliferation and oversupply of data was neither imagined nor a reality, affording women and families of childbearing age perhaps greater informational privacy and discretion around abortion and miscarriage care. Yet today, in light of ample and largely ungoverned opportunities to acquire potentially identifiable data from a wide variety of sources (eg, public websites, data brokers, and mobile apps), law enforcement access to identifiable or reidentifiable data to target women and families of childbearing age is easier than ever before. Complicating matters is the COVID-19 pandemic, which pushed many women and families of childbearing age to engage in family planning care virtually or online, ultimately boosting preexisting oversupplies of accessible yet sensitive digital-footprint data.

The literature is robust with commentaries and discussions on ethical, legal, and social considerations for consumer health data [7-17]. Although COVID-19 boosted online and digital engagement to accommodate social distancing and quarantine requirements worldwide [18-22], few studies to date have assessed how privacy-related experiences among research participants might shape their willingness to share their digital data with health researchers [19,23,24].

Amid growing concerns around corporate and law enforcement uses of digital data to target women and families of childbearing age, the recent Supreme Court ruling is likely to affect this population’s willingness to engage in health research if engagement involves the donation of their digital data. In this study, we explored the perspectives of a subpopulation of research participants of childbearing age regarding the health-relatedness of their digital data, their concerns about the use and sharing of personal data online, and their concerns about donating data from various sources to researchers today or in the future.

## Methods

### Survey Development

An 18-item electronic survey was developed using Qualtrics (Qualtrics Inc). Privacy-related survey items published by Seltzer et al [23] and validated and published by Doherty et al [25], as well as demographic-related survey items validated and published by Zhu et al [26], were selected, adapted, and revalidated for bias, relevance, and cognition among a convenience sample of 5 individuals who identified as both patients and health consumers. Based on the pilot participants’ feedback, the survey questions were refined to improve item quality and clarity and overall instrument clarity, appropriateness, and relevance.

### Study Population

In April 2021, the electronic survey was administered to adults (aged ≥18 years) registered in the ResearchMatch database [27] who agreed to be contacted to engage in the survey after receiving an informational electronic invitation letter via the ResearchMatch platform. ResearchMatch is a registry and collaborative project that is maintained at Vanderbilt University and overseen by the Vanderbilt University Institutional Review Board. ResearchMatch participants live within the US and Puerto Rico, are of all ages, races, and ethnicities, and comprise healthy volunteers as well as those living with medical conditions. Access to the ResearchMatch platform was provided through Ohio University.

### Survey Invitations and Distributions

Individuals were invited to participate in the survey regardless of health status, race, gender, or any other mutable or immutable characteristics. Survey participants were informed that their participation was entirely voluntary. No survey questions were mandatory, and participants were informed that they could skip any question at any time. Survey participants were welcomed to contact the research team at any time with any questions or concerns about the study. Reminders were sent up to 3 times to participants who began but had yet to complete and submit the survey within the study timeframe.

### Survey Incentives

Participants’ personal contact details were received only after participants agreed to participate in the survey. Those who completed and submitted the survey were offered a random chance to receive a US $250, $100, $50, or $25 gift card. A random selection tool was developed and deployed using Microsoft Excel (Microsoft Corp) to randomly select email addresses of survey participants and deliver the gift card incentives. Participant email addresses were deleted or destroyed to prevent reidentification at the conclusion of the study.

### Data Analysis

This study is part of a larger investigation to explore the privacy-related experiences and perspectives of US adults and their willingness to share digital data with researchers [24]. This analysis centers on a sample of ResearchMatch participants with a 100% response or completion rate (inclusive of completed surveys with items containing no responses). A Qualtrics tool was used to calculate an ideal survey sample size (n=384) based on the total ResearchMatch population (95% CI; 5% margin of error). Descriptive statistical analyses were conducted using Microsoft Excel. Manual queries (single layer, bottom-up topic modeling) were used to categorize illuminating quotes from free-text responses.

### Ethical Review and Oversight

This study was reviewed and approved by the Ohio University Institutional Review Board (20-E-457).

## Results

A total of 598 participants were invited to complete the survey, of which 470 initiated the survey (for a 79% response rate). Of the 470 participants who initiated the survey, 402 completed and submitted the survey (for an 86% completion rate) of which 47% (189/402) self-reported to be persons of childbearing age (18 to 50 years). Table 1 provides a brief summary of demographics for participant age and education level. The highest proportion of participants were aged between 21 and 40 years (132/189, 70%) and held a bachelor’s or master’s degree (127/189, 67%).

Participants were asked to indicate their level of agreement with statements that indicated the health-relatedness of various data sources (Table 2). Most participants agreed or strongly agreed that social media data (eg, Facebook, Twitter, and Instagram), email data, text message data, Google search history data, online purchase history data, electronic medical record data, fitness tracker or wearable data, credit card statement data, and genetic data are health-related. Most participants disagreed or strongly disagreed that music streaming data, Yelp review and rating data, ride-sharing history data, tax records and other income history data, voting history data, and geolocation data are health-related. Participants were largely neutral about the health-relatedness of Snapchat data.

Participants were asked to indicate whether they were concerned with 5 statements regarding the use and sharing of their personal data online (Table 3). Over 87% (164/189) of participants were concerned about fraud or abuse based on their personal information, online companies and websites sharing information with other parties without consent, and online companies and websites using information for purposes that are not explicitly stated in their privacy policies. Nearly 60% (111/189) of participants were concerned that information they share online with friends may be inappropriately disclosed by those friends to others. The lowest proportion of participants were concerned about people they know online not being who they say they are (87/189, 46%).

Lastly, participants were asked to share their concerns (in free text) about donating their electronic data to researchers today or in the future. Illuminating quotes were captured from participants who expressed concern with every statement shown in Table 3 regarding the use and sharing of their personal data online (59/189, 31%; Textbox 1). Illuminating quotes were lightly edited to address typos, organized, and categorized along the following themes: data use beyond scope of consent; exclusion from health care and insurance; government and corporate mistrust; and data confidentiality, security, and discretion.

**Table 1:** Survey participant education levels and age ranges (n=189).

Characteristics		Participants, n (%)
**Education level**		
	High school	7 (4)
	Some college/associate/trade school	37 (20)
	Bachelor’s degree	74 (39)
	Master’s degree	53 (28)
	Doctorate or other terminal degree	18 (10)
**Age range (years)**		
	Under 20	3 (2)
	21 to 30	59 (31)
	31 to 40	73 (39)
	41 to 50	54 (29)

**Table 2:** Respondents’ level of agreement with health-relatedness of various data sources (n=189).

Health-relatedness statements	Respondents, n (%)				
	Strongly disagree	Disagree	Neutral	Agree	Strongly agree
“Facebook data contain health-related information.”	13 (7)	32 (17)	46 (24)	86 (46)^a^	12 (6)^a^
“Twitter data contain health-related information.”	18 (10)	38 (20)	57 (30)	67 (35)^a^	9 (5)^a^
“Instagram data contain health-related information.”	18 (10)	36 (19)	52 (28)	72 (38)^a^	11 (6)^a^
“Snapchat data contain health-related information.”	23 (12)	48 (25)	65 (34)^a^	45 (24)	8 (4)
“Email accounts (Gmail, Yahoo, Comcast, Verizon, etc.) contain health-related information.”	14 (7)	16 (8)	34 (18)	93 (49)^a^	32 (17)^a^
“Text message data and phone call history contains health-related information.”	14 (7)	22 (12)	36 (19)	75 (40)^a^	42 (22)^a^
“Google search history contains health-related information.”	11 (6)	7 (4)	17 (9)	87 (46)^a^	67 (35)^a^
“Online purchase history (Amazon, Target, Google Buy, Ebay, etc.) contains health-related information.”	14 (7)	16 (8)	35 (19)	85 (45)^a^	39 (21)^a^
“Music streaming data (Spotify, Pandora, Apple Music, etc.) contains health-related information.”	55 (29)^a^	75 (40)^a^	37 (20)	18 (10)	4 (2)
“Electronic Medical Records contain health-related information.”	4 (2)	3 (2)	9 (5)	24 (13)^a^	149 (79)^a^
“Yelp reviews and ratings contain health-related information.”	30 (16)^a^	58 (31)^a^	57 (30)	41 (22)	3 (2)
“Ride-sharing history (Uber, Lyft, etc.) contains health-related information.”	39 (21)^a^	66 (35)^a^	52 (28)	27 (14)	5 (3)
“Fitness Tracker/Wearables history contains health-related information.”	3 (2)	2 (1)	15 (8)	74 (39)^a^	95 (50)^a^
“Tax records and other income history contains health-related information.”	39 (21)^a^	54 (29)^a^	48 (25)	40 (21)	8 (4)
“Credit card statements contain health-related information.”	32 (17)	40 (21)	39 (21)	63 (33)^a^	15 (8)^a^
“Voting history contains health-related information.”	74 (39)^a^	71 (38)^a^	30 (16)	12 (6)	2 (1)
“Geolocation (GPS from your phone or computer) data contains health-related information.”	27 (14)^a^	42 (22)^a^	54 (29)	54 (29)	12 (6)
“Genetic data (23andMe, etc.) contains health-related information.”	6 (3)	10 (5)	29 (15)	70 (37)^a^	74 (39)^a^

^a^These fields indicate highest proportions or skewed levels of agreement.

**Table 3:** Respondents’ concerns about the use and sharing of their personal information online (n=189).

Statements	Respondents, n (%)		
	Unsure	No	Yes
“Information I share with friends online may be inappropriately disclosed by them to others.”	20 (11)	58 (31)	111 (59)
“People who you only know from online are not who they say they are.”	52 (28)	50 (26)	87 (46)
“Other internet users might try to defraud you or abuse your personal information.”	8 (4)	17 (9)	164 (87)
“Online companies and websites might try and share your information to other parties without explicit consent.”	5 (3)	9 (5)	175 (93)
“Online companies and websites might use your information for purposes not explicitly stated in the privacy policy.”	9 (5)	14 (7)	166 (88)

**Illuminating quotes about donating electronic data to researchers from participants with concerns regarding the use and sharing of their personal information online.**

**Data use beyond scope of consent**

- “Biggest concern would be security of that donated data and being sure that it was being used as intended.”
- “My only concern is using the data for things other than health related issues.”

**Exclusion from health care and insurance**

- “I worry it could be used to deny me health or life insurance.”
- “Security of data, insurance coverage discrimination.”

**Government and corporate mistrust**

- “I would distrust my info being used by certain corporations.”
- “Privacy and info being sold or given to untrustworthy sources or companies.”
- “I don't think protections for data to be misused are strong enough in this country.”
- “After military service, I do not trust anyone with my health information.”
- “Data breaches or that the health data would be sold to corporations who would use that to market for me; that it would be collected by a government entity and used against me in any type of criminal or civil liability case.”
- “Releasing data of any kind raises my risk of adverse events, from identity theft to targeted biological warfare.”

**Data confidentiality, security, and discretion**

- “...potential for abuse...potential to find children people have lost custody of, like contacting them on Facebook instead of proper channels...harder to evade stalkers, etc.”
- “I’d like to keep my privacy. I’d feel really exposed. I’d have to know for sure that my data would be kept confidential and used for something good.”
- “I would have concerns regarding researchers keeping my information private, especially information regarding vaccine history.”
- “All of my concerns have to do with privacy. I would worry about how my information would be used and protected, and I would be especially sensitive about my genetic information.”
- “I have concerns regarding medical mistreatment due to past inaccurate diagnoses, and I am concerned about my health information being sold to advertisers.”
- “I realize all sorts of data is collected by many organizations. I generally attempt to avoid volunteering my health data, as it is extremely personal and identifiable.”
- “Don’t trust researchers’ ability to keep data secure. Anyone with a cyber security education or training could easily steal research data.”

## Discussion

### Principal Findings

This is the first and only study to explore the perspectives of a subpopulation of research participants of childbearing age about the health-relatedness of their digital data and accompanying privacy concerns. As mentioned, this assessment is part of a larger study to explore the privacy-related experiences and perspectives of US adults and their willingness to share real-world digital data with researchers [24]. Our findings indicate, in light of *Dobbs v Jackson* and other related events [28], a need to not only understand these phenomena, but also determine strategies and opportunities to educate research participants about how seemingly non–health-related data can become contextually relevant to abortion and miscarriage care.

For instance, a recent article published by the Brookings Institution [28] discussed how, recently, courts of law seeking to convict abortion seekers admitted data and evidence collected from individuals’ location data, text messages, and online activity. They highlighted 4 recent events:

- SafeGraph, a data curator company, sold phone location data generated by over 600 individuals who visited Planned Parenthood clinics, which led to the company issuing a public statement in June 2022 [29].
- In June 2022, the Center for Investigative Reporting and *The Markup* found that Facebook collected data on individuals visiting crisis pregnancy center websites [30].
- In 2017 in the US state of Mississippi, lawyers used evidence of a woman’s online search activity for abortion drugs in a court trial concerning her pregnancy loss [31].
- In 2015 in the US state of Indiana, a woman’s text message data to a friend about taking abortion pills was used to convict her in a court of law [32].

Our survey findings show that ResearchMatch participants are concerned about these very types of privacy-related events occurring in their lives. Our findings also indicate that ResearchMatch participants are likely correct in their judgments that social media data, email data, text message data, Google search history data, and online purchase history data are health-related in this context. Ironically, however, these events and our findings indicate that ResearchMatch participants are likely incorrect in their assumptions that ride-sharing history and geolocation data are not health-related. Moving forward, it will be critical to identify and implement resources to educate ResearchMatch participants and others about cases that demonstrate the contextual health-relatedness of ride-sharing history and geolocation data [33-37]. It will also be critical for data companies, app developers, and their collaborators to provide and support research and education about privacy-invasive software development kits and application programming interfaces that collect geolocation data.

Just prior to *Dobbs* (May 2022), in an attempt to educate the public about their privacy practices, SafeGraph released a public statement and formal letter in light of requests from legislators to explain how SafeGraph handled physical location and geolocation data [29]. The company explained their practice of aggregating data, implementing rigorous “differential privacy” [38], and removing device-level information and identifiers from data products prior to sharing them with third parties. They also stated that for their Patterns data product, which includes mobility data, they remove “aggregated, anonymized visit statistics for all businesses categorized by the North American Industry Classification System (NAICS) Code for family planning centers.” In the absence of a comprehensive federal data privacy law and amid an emerging patchwork of US state-level comprehensive privacy laws, companies like SafeGraph and others who track ride-sharing history and geolocation data should also collaborate to develop best practices for privacy and publicly endorse human rights protections for managing, processing, using, and sharing data that implicates businesses categorized by the North American Industry Classification System code as family planning centers (code 621410) [39]. By doing so, companies would not only provide transparency regarding their current practices to educate policy makers, regulators, and the public, but also cultivate strategies to improve their privacy practices in collaboration or consultation with neutral or third-party privacy experts.

In light of *Dobbs v Jackson* and related events [40], trust as well as data sharing and use transparency are necessary to recruit and retain participants in health research involving digital data. For instance, although a patchwork of comprehensive US state privacy laws [41] and a recent US executive order [42] exist to help preserve digital data privacy and protect access to reproductive health care services, respectively, further assurances are needed or should be made explicit to affirm the following: (1) the right to consent to the use, processing, storage, and deletion of data most perceived as health-related (ie, social media data, email data, text message data, online search history data, online purchase history data, electronic medical record data, fitness tracker or wearable data, credit card statement data, and genetic data); (2) protection against risk of fraud or abuse based on personal information by governments, corporations, health care providers, and insurers; (3) protection against data uses beyond the scope of consent or explicit statements in privacy policies; and (4) data confidentiality, security, and discretion.

Limitations should be noted when interpreting our results and directing future work. First, while our results provide important insights, they are merely descriptive due to our small sample size. Second, our analysis is not based on a representative sample of ResearchMatch participants or US adults of childbearing age. Most of our study participants reported high levels of education and most had at least a bachelor’s degree. Future work should endeavor to explore this topic in a more representative and larger sample of US adults of childbearing age.

Although income levels were not captured in this survey, educational level may serve as a proxy for income. As mentioned, most participants in this study had at least some college-level education or above, rendering it possible for them to have affordable and safe access to family planning services. According to the US Centers for Disease Control and Prevention [43], in 2019 women in their 20s accounted for the majority (56.9%) of those receiving abortion care nationally, though abortion ratios were highest among adolescents aged ≤19 years. Given the relatively low proportion of participants aged ≤20 years who completed our survey and given that the largest proportion of individuals engaged in the survey were aged 21 to 40 years, it is possible that the concerns and perspectives herein do not reflect those of adolescent women, who are most vulnerable to a lack of safe and affordable access to family planning services.

Our survey was developed on the basis of a recent systematic review and a survey study that both determined that age, income level, and education level were the strongest predictors of online or digital-footprint activity [44,45]. Thus, we collected these demographic variables in the survey but not sex, gender, or gender identity, as these variables were not suggested to be predictors of online or digital-footprint activity. Yet it should be noted that since the start of this survey, a more recent assessment of ResearchMatch participants has shown a large and growing number of participants (n=154,200) within the registry, with nearly 70% reporting as female and nearly 42% reporting as female parents. Thus, there is opportunity to explore the effect of gender on ResearchMatch participants’ views on the health-relatedness of their data.

Although our survey was conducted in April 2021, prior to the June 2022 US Supreme Court ruling in *Dobbs v Jackson*, ResearchMatch participants’ data privacy concerns may have been affected by COVID-19 and other major events that occurred during 2020 [23]. This is especially relevant given that recent studies show that individuals’ data privacy concerns were likely heightened during the COVID-19 pandemic, as privacy laws and regulations were relaxed to accommodate digital public health surveillance and remote health care [19,46]. Therefore, further research should explore how this recent US Supreme Court decision might impact female and female parent perspectives on the health-relatedness of their data and their concerns about donating data to researchers today or in the future.

### Conclusion

The US Supreme Court decision in *Dobbs v Jackson* has sharpened the focus on the ways in which populations perceive the health-relatedness of their data, on discretion around how data are used and processed, and on the organizations with whom data can be shared or disclosed. Recent uses of digital-footprint data to convict women in courts of law for engaging in abortion or undergoing certain types of miscarriage are both controversial and alarming. Therefore, it has become drastically important to understand the perspectives of health research participants of childbearing age regarding the health-relatedness of their digital-footprint data and their privacy concerns about sharing their digital-footprint data with researchers. After *Dobbs*, opportunities to educate research participants about the health-relatedness of their digital data are paramount. Determining strategies and privacy best practices for using digital-footprint data related to family planning with discretion should be a priority for companies, researchers, families, and other important stakeholders today and in the future.
